# A transfer learning approach to identify *Plasmodium* in microscopic images

**DOI:** 10.1371/journal.pcbi.1012327

**Published:** 2024-08-05

**Authors:** Jonathan da Silva Ramos, Ivo Henrique Provensi Vieira, Wan Song Rocha, Rosimar Pires Esquerdo, Carolina Yukari Veludo Watanabe, Fernando Berton Zanchi

**Affiliations:** 1 Computer Science Department, Federal University of Rondônia (DACC/UNIR), Porto Velho, Rondônia, Brazil; 2 Laboratório de Bioinformática e Química Medicinal, Fundação Oswaldo Cruz Rondônia, Porto Velho, Rondônia, Brazil; 3 Instituto Nacional de Epidemiologia na Amazônia Ocidental (INCT-EPIAMO), Porto Velho, Rondônia, Brazil; 4 Programa de Pós-Graduação em Biologia Experimental, Universidade Federal de Rondônia (PGBIOEXP/UNIR), Porto Velho, Rondônia, Brazil; Yale School of Public Health, UNITED STATES OF AMERICA

## Abstract

*Plasmodium* parasites cause Malaria disease, which remains a significant threat to global health, affecting 200 million people and causing 400,000 deaths yearly. *Plasmodium falciparum* and *Plasmodium vivax* remain the two main malaria species affecting humans. Identifying the malaria disease in blood smears requires years of expertise, even for highly trained specialists. Literature studies have been coping with the automatic identification and classification of malaria. However, several points must be addressed and investigated so these automatic methods can be used clinically in a Computer-aided Diagnosis (CAD) scenario. In this work, we assess the transfer learning approach by using well-known pre-trained deep learning architectures. We considered a database with 6222 Region of Interest (ROI), of which 6002 are from the Broad Bioimage Benchmark Collection (BBBC), and 220 were acquired locally by us at Fundação Oswaldo Cruz (FIOCRUZ) in Porto Velho Velho, Rondônia—Brazil, which is part of the legal Amazon. We exhaustively cross-validated the dataset using 100 distinct partitions with 80% train and 20% test for each considering circular ROIs (rough segmentation). Our experimental results show that DenseNet201 has a potential to identify *Plasmodium* parasites in ROIs (infected or uninfected) of microscopic images, achieving 99.41% AUC with a fast processing time. We further validated our results, showing that DenseNet201 was significantly better (99% confidence interval) than the other networks considered in the experiment. Our results support claiming that transfer learning with texture features potentially differentiates subjects with malaria, spotting those with *Plasmodium* even in Leukocytes images, which is a challenge. In Future work, we intend scale our approach by adding more data and developing a friendly user interface for CAD use. We aim at aiding the worldwide population and our local natives living nearby the legal Amazon’s rivers.

## Author summary

Malaria remains a significant threat to global health, affecting 200 million people and causing 400,000 deaths annually. Performing a reliable and efficient diagnosis still requires high-cost human training especially in needy and endemic regions. In Brazil, the main pathogen causing the cases is *Plasmodium vivax* with about 99% of malaria cases. Currently, in the era of artificial intelligence, we have at our disposal numerous algorithms with different variations applicable to the problem of pattern recognition in images. In this work, we performed the procedure known as supervised machine learning in which we used known data of cells infected with *Plasmodium vivax* including results of local data analysis. We used these data to train several networks and then applied them to real data in the form of training to verify the ability to identify *Plasmodium* in this new set. In our approach, we achieved in all approaches nothing less than 96% accuracy and in one case 99.41% accuracy. Thus, we fully demonstrate the possibility of using the methodology to develop a user-friendly interface to assist health agents in performing effective and efficient diagnosis even in hard-to-reach regions of our Amazon region.

## Introduction

Historically, Malaria has been a major human health concern caused by parasites from the *Plasmodium* genus, with *Plasmodium falciparum* and *Plasmodium vivax* causing the most of the cases [1]. Despite the decrease in malaria cases between 2000 and 2015, new cases started growing again in 2016, and the greatest increase took place between 2019 and 2020 amid the COVID-19 pandemic. According to the World Health Organization (WHO—https://www.who.int/), in 2020, almost half of the world’s population was at risk of contracting malaria. Thus, what was on a historical downward trend, went back up.

Several tropical diseases can manifest malaria-like symptoms, which can be misdiagnosed as malaria [2]. Relying solely on the symptoms presented by the patient may result in overdiagnosis and/or overtreatment [3]. Therefore, to diagnose malaria, various factors come into play: local circumstances; proficiency levels of laboratory staff; patient caseloads; and malaria prevalence in the target area [4]. Regardless of the diagnostic tool used, it is essential for it to yield rapid and accurate results to enable appropriate treatment of the patient [5].

The gold standard for malaria diagnosis is the microscopic technique. The analysis of blood smears in the microscope is commonly practiced and extensively documented. Moreover, it is a notably cost-effective method. However, the use of a microscope demands highly skilled specialists (good microscopists can identify 100–200 parasites per microliter) and access to well-equipped laboratories (availability of functional equipment, reagents, and an efficient quality control system) [3, 6, 7]. In addition, in remote regions with limited accessibility, performing microscopy can be extremely challenging [8].

Due to the wide range of variations in bright-field microscopy images, computational methods to aid in identification of *Plasmodium* parasites in blood smears remains largely unused. Recent advances in computer vision, especially in Deep Learning (DL) algorithms (Convolutional Neural Networks—CNN), have shown promising results in tasks involving the detection of abnormalities in medical images [9].

Previous attempts to automate, identify, and quantify *Plasmodium* parasites have not gained major traction, partly due to the difficulty of replication, comparison, and extension. Authors also rarely make their image sets available, which precludes replication of results and assessment of potential improvements. The lack of standard images or metrics to report results has impeded the field. In [10–14], the authors presented several DL methods with transfer-learning approaches for malaria detection. Generally, the authors use open-source databases and binarize the dataset into two classes (infected and non-infected). In general, studies that analyze only *P. vivax* discharge leukocyte cases. Unlike *P. falciparum*, *P. vivax* preferable infect young erythrocytes (red blood cell) [15, 16]. Therefore, *P. vivax* does not infect leukocyte (White Blood Cells), which may lead to false-positives.

They divided the train and test into a single partition and achieved promising results. The results vary, and some present accuracy closer to 100%, which encourages further studies in this context. They consider only well-segmented Red Blood Cells (RBCs) and analyze shape and texture features. However, without Leukocytes, the classification task becomes easier. In [17], the authors employed Darknet to classify avian malaria (*Plasmodium gallinaceum*) in four parasite stages. Although the dataset used was heavily unbalanced, the results were promising, achieving an average 99% AUC for each class with YOLOv3 and Darknet/Densenet201, which encourages the use o transfer learning for human *Plasmodium* parasite classification. Ikerionwu et al. [18] presented a systematic review of the machine and deep learning approaches to detect *Plasmodium* parasite in microscopic images. Authors affirm that human error is a major cause of inaccurate diagnostics in the traditional microscopy diagnosis method, which was later confirmed by Lee et al. [19].

Thus, a malaria diagnostic tool would be highly useful, and there is a need for more studies in machine and deep learning approaches to detect *Plasmodium* parasites in humans. Generally, DL works consider 90% for training and 10% for testing in a single partition. In our work, we considered all cases, including Leukocytes (harder to classify). We balance the dataset with 80% for training and 20% for testing on each of the exhaustive 100 distinct partitions. Thus, we avoid the possibility of randomly choosing a test set that leads to better results (biased).

Our main goal is to set a solid base to build up an application for Computer-aided Diagnosis (CAD) to differentiate between infected and non-infected Region of Interest (ROIs) in microscopic images. Thus, we aim at detecting *P. vivax* parasites in ROIs independently if the patient is symptomatic or not or what is the stage of the disease. In future work, we intend to investigate the aforementioned issues and achieve even better results by scaling our approach to gradually consider more samples.

We contribute in two main parts in this paper: (i) We provide a balanced dataset with local cases from Fundação Oswaldo Cruz (FIOCRUZ). Besides, we provide our codes for replication; (ii) We propose a workflow using transfer learning with circular cropping of the ROIs. Our cropping technique works as a rough segmentation, easing the CNNs process and improving classification. This ensures that if we apply a weak automatic segmentation (that does not fully segment each ROI,) the classification process can work.

## Results

We assessed three essential parts of our experimental design: First, we analyze the overall results considering our *Plasmodium* image database with representative infected and non-infected ROIs; Next, we assess the results using statistical testing. Finally, we present a run-time analysis.

### Overall results

**Fig 1:**
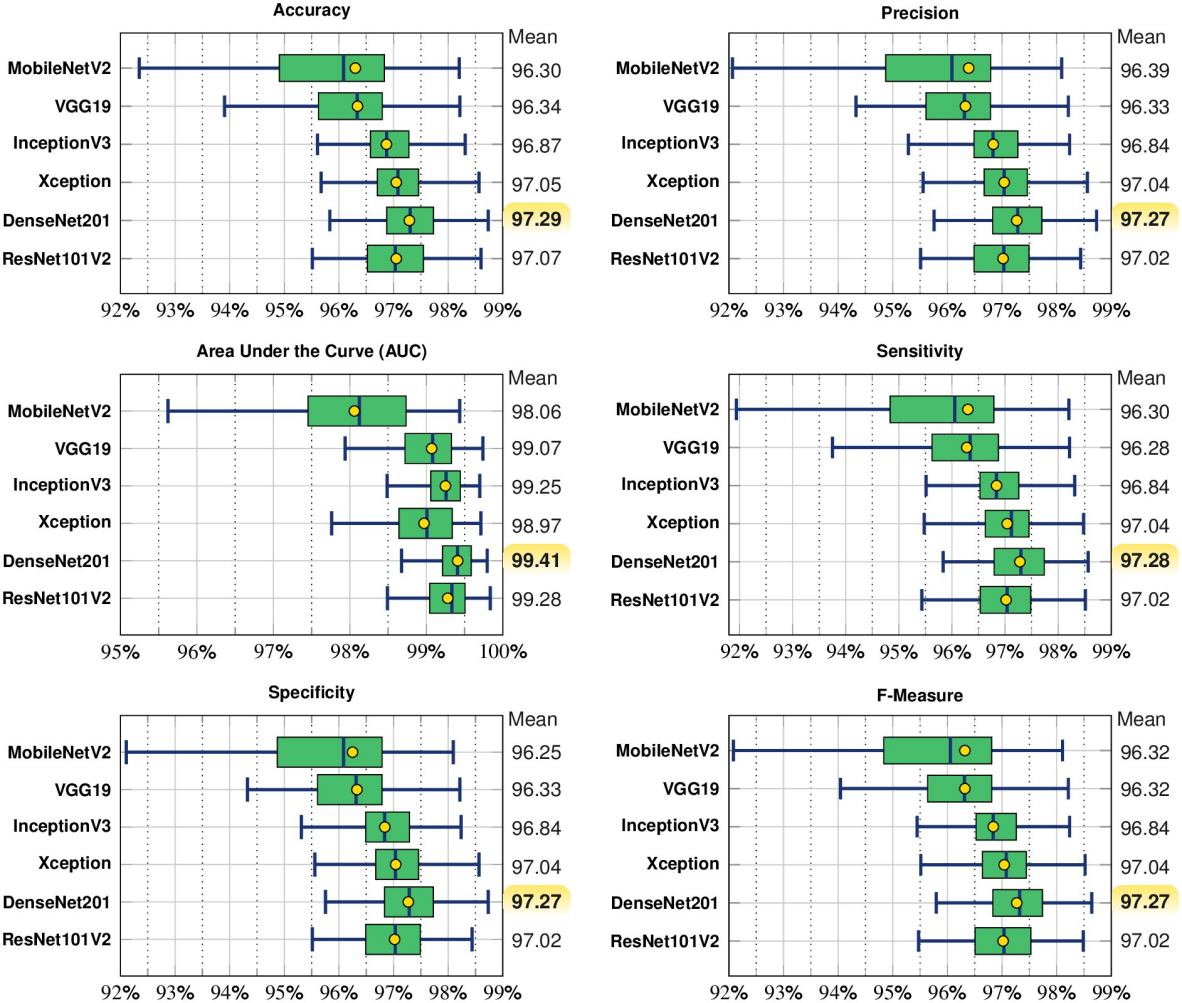
Overall results: Data distribution for each classifier for each measure considering the test set. The best average results are highlighted in yellow. The green area represents the Interquartile Range (IQR): 25th to the 75th percentile.

We analyze the data distribution of all network’s results, summarized in Fig 1. Overall, every approach presented promising results, averaging over 96% for each measure. DenseNet201 presented the highest average results for every measure with a more concise data distribution and closer to a normal distribution (visually). MobileNetV2 and VGG19 presented the lowest average results for all measures. InceptionV3, Xception, and ResNet101V2 presented similar results, however lower than DenseNet201. To further validate the results, in the next Section, we performed a statistical test validation to verify if the DenseNet201’s results are significantly better than the other networks.

### Statistical significance

We compared the 100 partition cross-validation results for every measure on each classification model. The Kolmogorov-Smirnov normality test [20, 21] showed that the data do not follow a normal distribution. Therefore, we employed the Wilcoxon test [22] to analyze whether the scores had statistically significant differences. We compared the method that presented the best classification results (DenseNet201) against the remaining methods.

Table 1 reports results for the Wilcoxon test, with a confidence interval of 99% and 95%, ticked in blue (✔) and magenta (✔), respectively. DenseNet201 presented significantly better results than the remaining methods most of the time with 99% confidence interval (✔). For 95% confidence interval (✔), DenseNet201 was significantly better than ResNet101V2 for the AUC and than Xception for the Specificity. However, note that the *p*-value was close to the 99% confidence interval (*p*-value < 0.0100). Thus, we can state, in general, that DenseNet201 presented significantly better results than the remaining networks considered in the study.

**Table 1:** Wilcoxon test *p*-values results: ✔ and ✔ indicate rejection of *H*_0_ with confidence interval of 99% and 95%, respectively. We considered DenseNet201 (data *a*) against the remaining methods (data *b*).

	Measures (*p*-values^a^)					
DenseNet201 (*a*) against (*b*)	Accuracy	Precision	AUC	Sensitivity	Specificity	F-Measure
MobileNetV2	✔ 0.0000	✔ 0.0000	✔ 0.0000	✔ 0.0000	✔ 0.0000	✔ 0.0000
VGG19	✔ 0.0000	✔ 0.0000	✔ 0.0000	✔ 0.0000	✔ 0.0000	✔ 0.0000
InceptionV3	✔ 0.0000	✔ 0.0000	✔ 0.0001	✔ 0.0000	✔ 0.0000	✔ 0.0000
Xception	✔ 0.0057	✔ 0.0098	✔ 0.0000	✔ 0.0061	✔ 0.0103	✔ 0.0069
ResNet101V2	✔ 0.0050	✔ 0.0063	✔ 0.0119	✔ 0.0031	✔ 0.0063	✔ 0.0050

Null hypothesis ***H***_0_: “*The median from data a **is not greater** than the median from data b*.”

We assess each network’s training and testing run-time to further validate our results in the next section.

### Run-time analysis

Comparing the Run-time in Table 2, MobileNetV2 presents the fastest processing time in test and training sets. However, as we aforementioned, MobileNetV2 did not present the best results. Although DenseNet201 did not present the fastest processing time for training/testing, it achieved the best results with a processing time closer to the other methods.

**Table 2:** Overall Run-time results (training and testing). ± represents the standard deviation.

	Run-time (Seconds)			
	*Training*		*Testing*	
Network	Mean	±	Mean	±
MobileNetV2	**112.55**	**54.68**	**1.07**	**0.27**
VGG19	257.98	95.16	1.71	0.50
InceptionV3	*111.04*	*28.16*	*1.69*	*0.26*
Xception	245.10	144.66	2.35	1.16
DenseNet201	206.12	42.62	3.88	0.65
ResNet101V2	225.48	59.51	2.91	0.14

DenseNet presents higher processing time due to the larger number of layers it contains (up to 152 layers), and for the very same reason. ResNet learns the residual representation functions instead of learning the signal representation directly [23], achieving better results in this case. Moreover, we highlight that the testing was performed considering 20% of the dataset, which means that, for DenseNet201, 1,244.4 ROIs were processed in 3.88 seconds. Thus, we can say that, on average, a single ROI was processed in 3.12 milliseconds by DenseNet201.

In this experiment, DenseNet201 presented better results at a higher processing time cost. DenseNet201 was projected to present better results running in dedicated servers, while, for example, MobileNet was projected to run on devices with lower processing power.

In an application context, such as a website service, processing an exam or a single ROI would be fast and acceptable. This processing time can also be reduced further with better computing resources. Therefore, we firmly believe our approach can be implemented and used in a real-case scenario without concern about the processing time.

## Discussion

This study applied transfer-learning (deep neural networks) to microscopic images of blood smears. We aimed at distinguishing infected and non-infected *Plasmodium vivax* parasites on each ROI. We assessed well-known pre-built network architecture using open-source images from BBBC. We added brand-new image cases, which we assembled locally in Porto Velho—RO/Brazil (legal Amazon) in partnership with FIOCRUZ.

Our results with balanced and cross-validated data divided into 100 partitions showed that Densenet201 has a potential to differentiate blood smears ROIs that are infected and non-infected with *P. vivax* parasites. Densenet201 presented an average AUC of 99.41% (with a 99% confidence interval), significantly better than the remaining methods. Generally, an AUC greater than 60% is considered acceptable in bio-medical sciences. However, an acceptable method in our experiment-wise should have an AUC higher than 90% [24]. Thus, our results were promising, and we achieved them by only preparing the ROIs in a circular manner and applying the transfer-learning approach. We strongly believe there is more to be explored in future works, including other malaria stains, more samples, etc.

One limitation of our study relates to the dataset preparation process. Every image used in our dataset was collected using blood smears stained with Giemsa reagent. We can not guarantee it would work with a different stained reagent. We intend to experiment with it in other studies. Another limitation regards the regions of interest (ROIs). In our work, the ROIS presented a preliminary rough segmentation predominantly featuring a circular shape. In this way, the network uses only texture features to analyze each case. However, it is worth mentioning that, in the literature, automatic approaches, including YOLO (https://pjreddie.com/darknet/yolo/), have shown promising results in identifying ROIs within microscopic images 145 domain.

Since several microscopes and cameras were used in inter and intra-datasets, we firmly believe that our proposed approach can be used in a real-case scenario, allowing a second opinion, thus, aiding the specialists. In future works, we intend to develop an application to perform second opinions in a friendly user interface. Moreover, we intend to assess the efficacy of our approach when the ROIs present parasites with unusual morphology. Moreover, we intend to robust our approach to work with different *Plasmodium* species as well as identifying different blood stages. We also would like to highlight that more images can be added, and the network can be retrained to achieve even more precise results.

## Materials and methods

**Fig 2:**
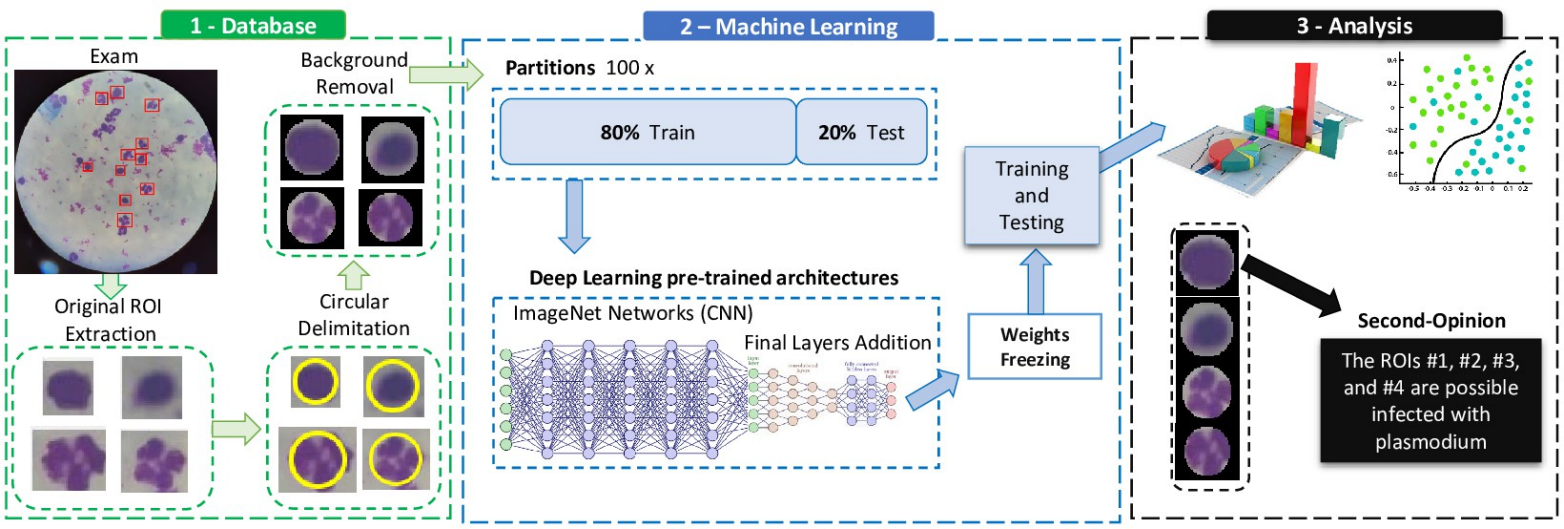
Workflow employed in this work to identify *Plasmodium*-infected ROIs.

This Section presents the proposed approach and experiments’ materials and methods. Fig 2 illustrates the workflow of this work, which we have divided into three main parts.

We briefly discuss each part below, exploring each in detail in the corresponding Section. First, we considered a representative database in Section Database, comprising 1.176 exams and 6.112 samples (ROIs). Then, we created 100 distinct partitions for cross-validation (Section Cross-validation). After that, in Section Pre-trained deep learning architectures, we employed several DL pre-trained architectures. Finally, in Section Measures and computing resources we describe well-known measures we consider in our experimental design and computational resources.

We made available all codes and ROIs used in our study at [25].

### Database

We considered a representative database/exams, which we assembled by combining the Broad Bioimage Benchmark Collection (BBBC—https://bbbc.broadinstitute.org/BBBC041) [26] with images from the Fundação Oswaldo Cruz (FIOCRUZ) in Porto Velho, Rondônia—Brazil. In both datasets, only *P. vivax* malaria strains exist. It is one of the most common cases in America. For this reason, more data samples of this strain are available, and it is a good starting point for automatic malaria analysis. An expert malaria researcher annotated all cell objects (boxed and labeled), including red blood cells and leukocytes. Moreover, bounding boxes were marked by an expert technician. The BBBC database contains 1364 images (approximately 80,000 cells) from Brazil and Southeast Asia. The data had a heavy imbalance towards uninfected cells versus infected cells, making up over 95% of uninfected cells. Among the infected cells ROIs, there are different stages of *P. vivax* parasites, including gametocyte, ring, trophozoite, and schizont. Therefore, we balanced the BBBC database by randomly selecting 3001 non-infected cells (ROIs). We took the number of infected cells for each image and randomly selected the same number of non-infected cases from that same image. We divided the labeled cells (ROIs) into two categories(1) healthy cells (3.001 cases) and (2) infected cells (3.001 cases).

Table 3 summarizes the final assembled database. For the FIOCRUZ database, we did not consider exams that presented infected and non-infected ROI-cases simultaneously. We segregated the exams by considering only positive cases from one exam and negative cases from another. Blood smears were stained with Giemsa reagent for both databases, and images were taken using default microscopic parameters (10x eyepiece) for *Plasmodium* analysis. However, several microscopes and cameras were used in inter and intra-datasets for the BBBC dataset. For the FIOCRUZ dataset, a single microscope was used, and only the focus parameters varied, which is a common practice when acquiring this kind of image. Therefore, since the acquisition protocol is the same worldwide for the acquisition of blood smears images, we did not include any bias by adding our local images. Every image is in Red, Green, and Blue—RGB format and always contain 3 channels. Overall, the images present a magnification of 100x in the objective lens and a variation of 7x to 10x in the ocular lens, yielding an average pixel resolution of 1, 240 × 1, 645, while the ROI sizes present average pixel resolution of 110 × 110.

**Table 3:** Summary of our assembled exams database.

	Resolution			Total of			
Database	Exam	Bounding box	Final ROI	Exams	infected cells	uninfected cells	cells (ROIs)
BBBC	1, 219 × 1, 635 ±55 × 104	123 × 124 ±24 × 24	110 × 110 ±17 × 17	1148	3001	3001	6002
FIOCRUZ	2, 156 × 2, 090 ±1, 022 × 995	93 × 102 ±52 × 56	89 × 89 ±47 × 46	27	110	110	220
**Overall**	1, 240 × 1, 645 ±214 × 193	122 × 124 ±26 × 26	110 × 110 ±20 × 20	1175	3111	3111	**6222**

Since the overall dataset perfectly balances positive and negative cases, we did not consider any data augmentation approach to avoid bias in the final results. We made available all datasets codes and results achieved in our work (https://github.com/JonathanRamos/PlasmodiumAI).

#### ROI cropping in circular shape

**Fig 3:**
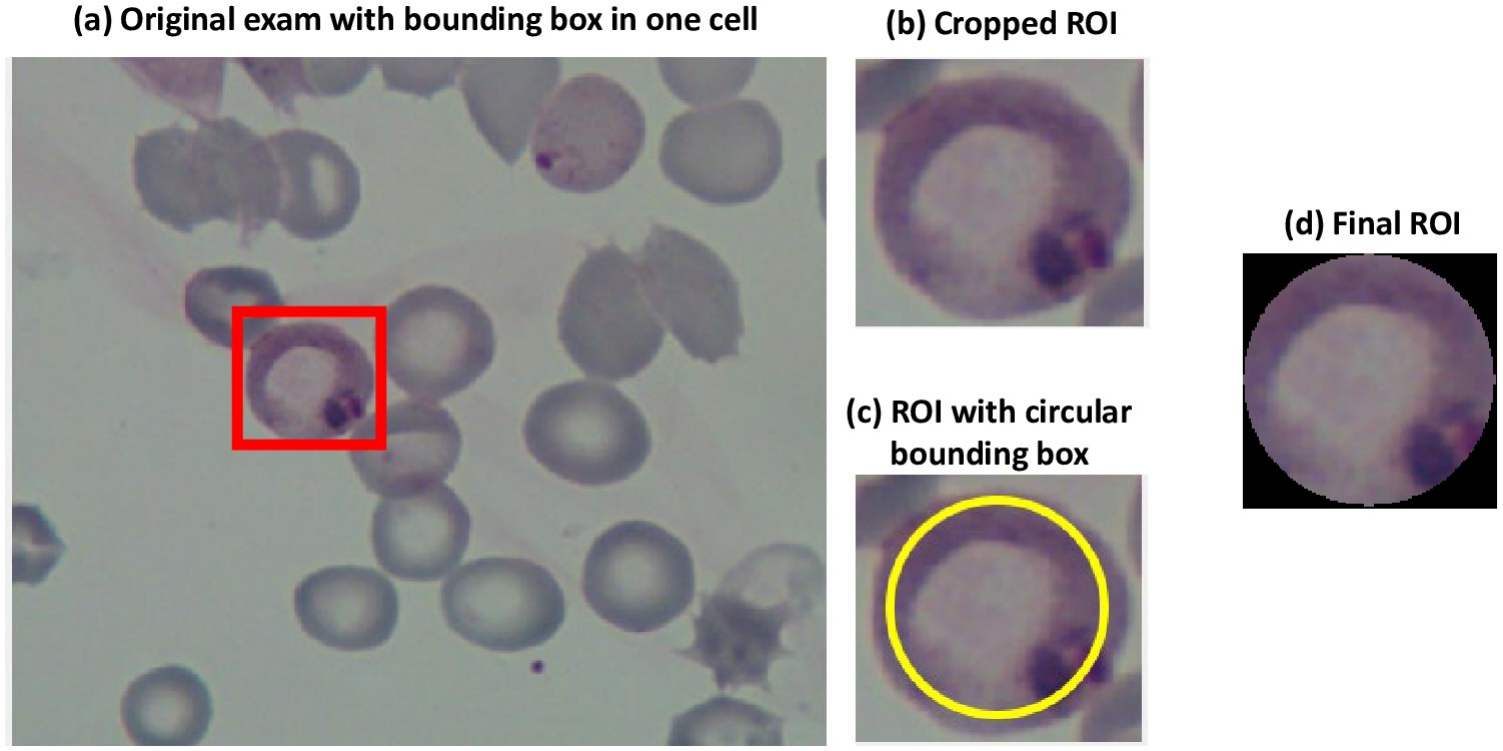
ROI cropping example.

We cropped the ROIs as illustrated in Fig 3. First, in (a), each exam presents a bounding box for each marked blood cell so that the ROI can be cropped as in (b). Next, we discharged irrelevant information around the cell for better analysis by applying circular cropping, as (c). We produce the final ROI in (d) by cropping the original ROI using a circular mask that considers 90% of the original ROI size (in yellow in Fig 3(c)). Although it may not provide optimal accuracy compared to automatic segmentation methods, it works as a rough segmentation, which is a good starting point. Thus, easing the classification process and discharging shape features, and working only with texture and color features.

Note that the number of pixels considered in each ROI may vary due to the distinct size of the original bounding boxes. Overall, the original ROIs present a resolution of 122 × 124 ± 26 × 26. In comparison, our final ROIs present a resolution of 110 × 110 ± 20 × 20, both in RGB format (3 channels) as described in Table 3. The ROIs are re-scaled for each transfer learning architecture to fit into the minimum ROI size required by each network by padding black pixels. We want to highlight that we did not process the ROIs with any kind of color/illumination normalization or interpolation. Therefore, we allow generalizability of the acquisition process.

### Cross-validation

We performed a cross-validation considering 80% of the samples for training and 20% for the test. We divided (partitioned) the data in this way 100 times, generating 100 unique partitions. In each partition, we were careful to not include ROIs from the same exam in test and train simultaneously.

### Pre-trained deep learning architectures

We have used several Deep Learning (Convolutional Neural Networks—CNNs) approaches well suited to classify medical images [27]. This paper presents the results for six promising pre-built DL architectures from Tensorflow/Keras library, namely MobileNetV2, VGG19, InceptionV3, Xception, DenseNet201, and ResNet101V2. To apply the transfer-learning approach for every network, we considered the pre-trained weights of ImageNet (https://keras.io/api/applications/) for those networks and applied a weight freezing technique.

Initially, we receive the ROIS as input of size 110 × 110 ± 20 × 20 (Table 3), and then resize them to 128 × 128 by cropping/padding black pixels without using any interpolation. The third dimension represents the Red, Blue, and Green channels (RGB). Then, we considered a batch size of 32 and a learning rate of 1e^−5^ with a max of 100 epochs. Finally, we added new layers for every network after the last layer of the default architectures as follows. First, a Global Average Pooling 2D followed by a dense layer using the Rectified Linear Unit (ReLU) activation. Then, a Dropout layer with a final dense layer of 2 units with softmax activation. We used a dense layer of 128 units for every network, a DropOut value of 0.3, and a freeze percentage of 30% Moreover, aimed to avoid over-fitting, we used early stopping.

### Measures and computing resources

We evaluated the classification quality with well-established measures: Accuracy, Precision, Sensitivity, Specificity, F-Measure (FM), and Area Under the Curve (AUC) [28]. We also measured the results’ statistical significance.

We performed the experiments on an RYZEN(R) 7 5700G CPU, 32GB machine, NVIDIA RTX2060 6GB RAM GPU. We employed Python 3.9.0 with Tensorflow/Keras V2.5.0 and Miniconda with CUDA toolkit (https://anaconda.org/anaconda/cudatoolkit) V11.2.2 on a Windows 11 Operating System. We made available the source code of all experiments in [25].

## References

[1] Cowman AF, Healer J, Marapana D, Marsh K. Malaria: Biology and Disease. Cell. 2016;167(3):610–624. doi: 10.1016/j.cell.2016.07.055 27768886

[2] Oladosu OO, Oyibo W. Overdiagnosis and Overtreatment of Malaria in Children That Presented with Fever in Lagos, Nigeria. International Scholarly Research Notices. 2013;2013:1–6.

[3] Gitta B, Kilian N. Diagnosis of malaria parasites Plasmodium spp. In endemic areas: Current strategies for an ancient disease. Bioessays. 2020;42(1):e1900138. doi: 10.1002/bies.201900138 31830324

[4] Moonasar D, Goga AE, Frean J, Kruger P, Chandramohan D. An exploratory study of factors that affect the performance and usage of rapid diagnostic tests for malaria in the Limpopo Province, South Africa. Malaria Journal. 2007;6(1):74. doi: 10.1186/1475-2875-6-74 17543127 PMC1891308

[5] Wongsrichanalai C, Barcus MJ, Muth S, Sutamihardja A, Wernsdorfer WH. A review of malaria diagnostic tools: microscopy and rapid diagnostic test (RDT). Am J Trop Med Hyg. 2007;77(6 Suppl):119–127. doi: 10.4269/ajtmh.2007.77.119 18165483

[6] Heide J, Vaughan KC, Sette A, Jacobs T, Schulze Zur Wiesch J. -Specific CD8+ T Cell Epitopes. Front Immunol. 2019;10:397. doi: 10.3389/fimmu.2019.00397 30949162 PMC6438266

[7] Ngasala B, Bushukatale S. Evaluation of malaria microscopy diagnostic performance at private health facilities in Tanzania. Malaria Journal. 2019;18(1). doi: 10.1186/s12936-019-2998-1 31771572 PMC6880513

[8] Wangdi K, Pasaribu AP, Clements ACA. Addressing hard-to-reach populations for achieving malaria elimination in the Asia Pacific Malaria Elimination Network countries. Asia; the Pacific Policy Studies. 2021;8(2):176–188. doi: 10.1002/app5.315

[9] Bouwmans T, Javed S, Sultana M, Jung SK. Deep neural network concepts for background subtraction:A systematic review and comparative evaluation. Neural Networks. 2019;117:8–66. doi: 10.1016/j.neunet.2019.04.024 31129491

[10] Siłka W, Wieczorek M, Siłka J, Woźniak M. Malaria Detection Using Advanced Deep Learning Architecture. Sensors. 2023;23(3). doi: 10.3390/s23031501 36772541 PMC9921611

[11] Alnussairi MHD, İbrahim AA. Malaria parasite detection using deep learning algorithms based on (CNNs) technique. Computers and Electrical Engineering. 2022;103:108316. doi: 10.1016/j.compeleceng.2022.108316

[12] Jameela T, Athota K, Singh N, Gunjan VK, Kahali S. Deep Learning and Transfer Learning for Malaria Detection. Computational Intelligence and Neuroscience. 2022;2022:14. doi: 10.1155/2022/2221728 35814548 PMC9259269

[13] Hemachandran K, Alasiry A, Marzougui M, Ganie SM, Pise AA, Alouane MTH, et al. Performance Analysis of Deep Learning Algorithms in Diagnosis of Malaria Disease. Diagnostics. 2023;13(3). doi: 10.3390/diagnostics13030534 36766640 PMC9914762

[14] Rahman A, Zunair H, Reme TR, Rahman MS, Mahdy MRC. A comparative analysis of deep learning architectures on high variation malaria parasite classification dataset. Tissue and Cell. 2021;69:101473. doi: 10.1016/j.tice.2020.101473 33465520

[15] Golenda CF, Li J, Rosenberg R. Continuous in vitro propagation of the malaria parasite Plasmodium vivax. Proc Natl Acad Sci U S A. 1997;94(13):6786–6791. doi: 10.1073/pnas.94.13.6786 9192643 PMC21236

[16] Udomsangpetch R, Somsri S, Panichakul T, Chotivanich K, Sirichaisinthop J, Yang Z, et al. Short-term in vitro culture of field isolates of Plasmodium vivax using umbilical cord blood. 10.1016/j.parint.2006.12.005 17254835

[17] Kittichai V, Kaewthamasorn M, Thanee S, Jomtarak R, Klanboot K, Naing KM, et al. Classification for avian malaria parasite Plasmodium gallinaceum blood stages by using deep convolutional neural networks. Scientific Reports. 2021;11(1). doi: 10.1038/s41598-021-96475-5 34413434 PMC8376898

[18] Ikerionwu C, Ugwuishiwu C, Okpala I, James I, Okoronkwo M, Nnadi C, et al. Application of machine and deep learning algorithms in optical microscopic detection of Plasmodium: A malaria diagnostic tool for the future. Photodiagnosis and Photodynamic Therapy. 2022;40:103198. doi: 10.1016/j.pdpdt.2022.103198 36379305

[19] Lee RM, Eisenman LR, Khuon S, Aaron JS, Chew TL. Believing is seeing—the deceptive influence of bias in quantitative microscopy. Journal of Cell Science. 2024;137(1). doi: 10.1242/jcs.261567 38197776

[20] Massey FJ. The Kolmogorov-Smirnov test for goodness of fit. Journal of the American Statistical Association. 1951;46(253):68–78. doi: 10.1080/01621459.1951.10500769

[21] King AP, Eckersley RJ. Chapter 6—Inferential Statistics III: Nonparametric Hypothesis Testing. In: King AP, Eckersley RJ, editors. Statistics for Biomedical Engineers and Scientists. Academic Press; 2019. p. 119–145.

[22] Wilcoxon F, Katti SK, Wilcox RA. Critical values and probability levels for the Wilcoxon rank sum test and the Wilcoxon signed rank test. Selected Tables in Mathematical Statistics. 1970;1:171–259.

[23] He K, Zhang X, Ren S, Sun J. Deep Residual Learning for Image Recognition. In: 2016 IEEE Conference on Computer Vision and Pattern Recognition (CVPR); 2016. p. 770–778.

[24] Hajian-Tilaki K. Receiver Operating Characteristic (ROC) Curve Analysis for Medical Diagnostic Test Evaluation. Caspian J Intern Med. 2013;4(2):627–635. 24009950 PMC3755824

[25] Ramos JS. Plasmodium Identification Using Transfer Learning; 2023. Available from: https://github.com/JonathanRamos/PlasmodiumAnalysis.

[26] Hung J, Lopes SCP, Nery OA, Nosten F, Ferreira MU, Duraisingh MT, et al. Applying Faster R-CNN for object detection on malaria images. Conf Comput Vis Pattern Recognit Workshops. 2017;2017:808–813. doi: 10.1109/cvprw.2017.112 34938593 PMC8691760

[27] Roberts M, et al. Common pitfalls and recommendations for using machine learning to detect and prognosticate for COVID-19 using chest radiographs and CT scans; 2021.

[28] Sokolova M, Lapalme G. A systematic analysis of performance measures for classification tasks. Information Processing & Management. 2009;45(4):427–437. doi: 10.1016/j.ipm.2009.03.002

